# Evaluation of MDCK Cell-Derived Influenza H7N9 Vaccine Candidates in Ferrets

**DOI:** 10.1371/journal.pone.0120793

**Published:** 2015-03-23

**Authors:** Min-Yuan Chia, Alan Yung-Chih Hu, Yu-Fen Tseng, Tsai-Chuan Weng, Chia-Chun Lai, Jun-Yang Lin, Po-Ling Chen, Ya-Fang Wang, Sin-Ru Chao, Jui-Yuan Chang, Yi-Shiuh Hwang, Chia-Tsui Yeh, Cheng-Ping Yu, Yee-Chun Chen, Ih-Jen Su, Min-Shi Lee

**Affiliations:** 1 National Institute of Infectious Diseases and Vaccinology, National Health Research Institutes, Zhunan, Taiwan; 2 Institute of Preventive Medicine, National Defense Medical Center, Taipei, Taiwan; University of Georgia, UNITED STATES

## Abstract

Avian-origin influenza A (H7N9) viruses emerged as human pathogens in China in early 2013 and have killed >100 persons. Influenza vaccines are mainly manufactured using egg-based technology which could not meet the surging demand during influenza pandemics. In this study, we evaluated cell-based influenza H7N9 vaccines in ferrets. An egg-derived influenza H7N9 reassortant vaccine virus was adapted in MDCK cells. Influenza H7N9 whole virus vaccine antigen was manufactured using a microcarrier-based culture system. Immunogenicity and protection of the vaccine candidates with three different formulations (300μg aluminum hydroxide, 1.5μg HA, and 1.5μg HA plus 300μg aluminum hydroxide) were evaluated in ferrets. In ferrets receiving two doses of vaccination, geometric mean titers of hemagglutination (HA) inhibition and neutralizing antibodies were <10 and <40 for the control group (adjuvant only), 17 and 80 for the unadjuvanted (HA only) group, and 190 and 640 for the adjuvanted group (HA plus adjuvant), respectively. After challenge with wild-type influenza H7N9 viruses, virus titers in respiratory tracts of the adjuvanted group were significantly lower than that in the control, and unadjuvanted groups. MDCK cell-derived influenza H7N9 whole virus vaccine candidate is immunogenic and protective in ferrets and clinical development is highly warranted.

## Introduction

Avian-origin influenza A (H7N9) viruses emerged as human pathogens in China in early 2013 and 450 laboratory-confirmed human cases, including 165 deaths, have been reported to the World Health Organization (WHO) by 27 June 2014, including 10, 4 and 1 imported cases detected in Hong Kong, Taiwan and Malaysia, respectively [1]. The cases occurred in an initial wave from February to May 2013 (n = 133), and a second wave of epidemics has reemerged from June 2013 to June 2014 (n = 317) [1]. Laboratory analysis of influenza H7N9 viruses isolated from human, animal and environmental samples during the first and second waves indicates that the hemagglutinin (HA) and neuraminidase (NA) genes of the viruses remain similar, and all viruses are antigenically close to A/Anhui/1/2013 (H7N9) virus which was recommended for vaccine development by the WHO. Since much remain unknown about this virus, such as 1) the animal reservoirs in which it is circulating, 2) the main exposures and routes of human transmission, and 3) prevalence of this virus in human and animal populations in endemic areas, it is important to continue strengthening surveillance and national pandemic preparedness including stockpile of antivirals and vaccine development, especially for Asian countries close to China [2–7].

Current seasonal influenza vaccines are mainly manufactured using egg-based technology. However, the egg-based technology could not meet the surging global demand during an influenza pandemic as proved in the 2009 H1N1 pandemic [8]. Therefore, it is desirable to establish cell-based influenza vaccine production platform for pandemic preparedness [9]. Since A/Anhui/1/2013(H7N9)-like viruses were recommended for vaccine development in April 2013, several egg-derived high growth reassortant vaccine viruses have been generated by the WHO reference laboratories. However, the WHO reference laboratories have not generated cell-derived high growth vaccine viruses, which is the critical step to initiate production of cell-based influenza vaccines. In this study, we adapted the egg-derived influenza H7N9 vaccine viruses in mammalian cells to generate cell-derived vaccine viruses, manufactured influenza whole virus vaccines in a microcarrier cell culture system, and evaluate immunogenicity and protection of the vaccine candidates in ferrets. Based on clinical studies of the influenza H5N1 and H7N1 vaccines, whole virus vaccine antigens are more immunogenic in naive populations than split and subunit vaccine antigens [10, 11].

## Materials and Methods

### Virus, cells, and medium

An egg-derived influenza H7N9 reassortant vaccine virus (NIBRG-268) was generated using reverse genetics by the UK National Institute of Biological Standard and Control (NIBSC) and supplied to National Health Research Institutes (NHRI), Taiwan. The NIBRG-268 virus contains six internal genes of the egg-adapted high-growth A/PR8 virus and two surface protein genes (HA and NA) of A/Anhui/1/2013(H7N9). At NHRI, the NIBRG-268 virus could not grow well initially in Madin-Darby canine kidney (MDCK) cells (∼10^5^ TCID_50_/ml). Therefore, we serially passed the NIBRG-268 virus in MDCK cells to increase its growth efficiency. MDCK cells (ATCC CCL-34) were purchased from the Food Industry Research and Development Institute, Hsinchu, Taiwan. MDCK cells were grown using DMEM (GibcoBRL) plus 5% fetal bovine serum (Moregate) to generate master and working cell banks following cGMP guidelines and have been characterized to fulfill the requirements for continuous cell lines used for manufacture of biological products [12–14]. For vaccine production, serum-free medium (OptiPro, Life Technologies) was used for cell growth and OptiPro supplemented with 2 μg/ml of TPCK-trypsin (Sigma) was used for virus replication.

### Virus titration

HA titrations were conducted in 96-well microplates using turkey red blood cells (RBC) following the standard technique [15]. Virus infectious titers were measured using the 50% tissue culture infectious doses (TCID_50_) assay based on cytopathic effect and plaque assay based on plaque forming unit (PFU) in MDCK cells [15]. A positive control with pre-specified acceptable range was included for conducting HA, TCID_50_, and plaque assays.

### Production of vaccine antigens in microcarrier-based cell cultures

In general, the regulatory hurdle of inactivated influenza vaccines is lower than that of live-attenuated influenza vaccines. In addition, historical data have shown that inactivated whole virus antigens have been more immunogenic in naïve populations than split and subunit vaccine antigens [10, 11, 16–21]. Therefore, we decided to develop inactivated whole virus as vaccine candidates. Cytodex 1 microcarriers (GE Healthcare) were hydrated, autoclaved, and preconditioned according to the manufacturer’s instructions [12, 13]. When the cells were grown confluent on microcarriers, microcarriers were sampled to count cell density and the vaccine viruses were added to infect cells. Supernatants of virus growth were clarified and inactivated with formaldehyde. Virus inactivation was confirmed with TCID_50_ and plaque assays. After inactivation and concentration, downstream purification processes comprising of size-exclusion chromatography (Capto Q, GE Healthcare), anion-exchange chromatography (Capto Core, GE Healthcare), and membrane filtration (Hydrosate, GE Healthcare) were used. The HA protein concentrations of purified inactivated vaccine antigens were measured using the standard single radial diffusion (SRD) assay with the standard antigen and antiserum obtained from the US FDA [22].

### Electron microscopy analysis

Viral particles were deposited on a carbon-coated 200 mesh copper grid for 1 min at room temperature and then the copper grid was stained with 2% phosphotungstic acid solution for 1 min. The stained grid was dried for 1 day at room temperature and observed under a JEM 1200EX transmission electron microscopy [23].

### Vaccine efficacy in ferrets

Four- to six-month-old female ferrets were obtained through a laboratory breeding program at Institute of Preventive Medicine, National Defense Medical Center and confirmed to be H7N9 seronegative by hemagglutination inhibition assay. Prior to the animal study, microchip capable of measuring the temperature was implanted beneath the skin between the shoulder blades. Ferrets were numbered and housed separately in animal biosafety level 3 (ABSL3) laboratory with individual temperature control at 22–24°C and 55–60% relative humidity throughout the experiment.

Ferrets were immunized intramuscularly with 2 doses of vaccine antigen at a 2-week interval. Three treatment groups (4 ferrets per group) with vaccines containing different HA and adjuvant dosages were compared, including 1) 300μg Al(OH)_3_, 2) 1.5μg HA, and 3) 1.5μg HA+300μg Al(OH)_3_. Solicited local symptoms at injection site were evaluated during the 7 days postvaccination period after each dose of H7N9 vaccine. Sera were collected at days 0 (prevaccination), 14 (post dose 1), and 21 (1 week post dose 2) for measuring HI and neutralizing antibody titers. At Day 28, ferrets were intranasally inoculated with wild-type influenza A/Anhui/1/2013 (H7N9) viruses (10^7^ TCID_50_/0.5 ml). Clinical signs of body weight and temperature were monitored daily for 7 days post challenge (DPC). At Day 31 (3 DPC) and 35 (7 DPC), two ferrets were sacrificed respectively to collect multiple organs (nasal turbinate, upper respiratory tract, lower respiratory tract, lung, and hilar lymph node) for measuring viral RNA copies by real-time polymerase chain reaction (qPCR). Live infectious virus titers were further quantified for nasal turbinate and other organs with significant viral RNA copies (>3 log_10_ RNA copies/mg). In pilot studies, ferrets infected with wild-type influenza A/Anhui/1/2013 (H7N9) viruses did not develop clinical symptoms and only shed viruses in respiratory tract within 7 days post inoculation. Therefore, ferrets were only followed up for 7 days post inoculation in the vaccine protection study.

### Real-time RT-PCR

One hundred milligram of tissue sample was homogenized mechanically in DMEM to make a 10% suspension. After vigorous shaking, the samples were centrifuged at 8000 × *g* for 15 min at 4°C. The viral RNA was extracted from supernatants by QIAamp Viral RNA Kit (Qiagen) according to the manufacturer’s instruction. Each RNA sample was then reversely transcribed using the Random primer (Bioline) and the HiScript I Reverse Transcripase (Bionovas) according to the manufacturer’s instructions. Copy numbers of influenza Matrix (M) gene were determined by a 7500 Real Time PCR System (Applied Biosystems) with Power SYBR Green PCR Master Mix (Applied Biosystems) as described previously [6, 24]. Positive and negative reference samples were tested along with the unknown samples and each sample was tested in duplicate.

### Pathological examination and immunohistochemical (IHC) staining

All ferrets were sacrificed at designated time points to collect multi organs for viral titer determination and pathological examination. Ferrets were anesthetized by injection with 7.5 mg/kg Zoletil 50 (Virbac) and inhalation with Isoflurane (Panion & Bf Biotech) and euthanized by taking total amount of blood from heart. Tissue specimens form nasal turbinate, upper respiratory tract, lower respiratory tract, lung, and hilar lymph node were collected for further analysis. Excised tissues of ferret organs were stored at −70°C until titration or fixed with 10% phosphate-buffered formalin at room temperature for pathological examination. Tissues were trimmed, dehydrated, embedded, and cut into 5μm-thick sections in procession. One section from each tissue was stained by a standard histochemistry staining procedure with hematoxylin and eosin (Sigma), whereas another one was deparaffinized with xyline and rehydrated through a series graded alcohol solutions for standard IHC staining with a mouse anti-nucleoprotein (NP) monoclonal antibody (provided by Animal Technology Institute Taiwan). Detection was performed by UltraVision Quanto detection system HRP DAB (Thermo Scientific) according to the manufacturer’s instructions. Sections were counterstained in Mayer’s hematoxylin (ScyTeK Laboratories) and mounted by Surgipath micromount medium (Leica Biosystems).

### Serological assays

Serum HI antibody titers were measured using turkey red blood cells and 4 HA unit of vaccine virus antigen following WHO’s standard procedures [15]. Serum neutralizing antibody titers were determined using MDCK cells and are expressed as the reciprocal of the highest dilution of serum that gave 50% neutralization of 100 TCID_50_ of the vaccine virus following the WHO standard procedures [15]. Starting dilutions are 1:10 in the HI assay and 1:40 in the neutralization assay, so undetectable antibody titers were assigned to 5 and 20, respectively. Seroconversion is defined as conversion from seronegative (antibody titers <10 in the HA assay and <40 in the Nt assay) to seropositive (antibody titers ≥20 in the HA assay and ≥80 in the Nt assay). A positive control serum with pre-specified acceptable range was included for HI and neutralization tests.

### Statistical analysis

In HI and neutralization tests, antibody titers were log-transformed to calculate geometric mean titers (GMT) and their 95% confidence intervals (95% CI). Differences in HI and neutralization titers were tested for statistical significance by using the unpaired Student *t* test. For infection study, changes in body temperatures and weights were measured by calculating percentage compared with baseline (prevaccination) in each ferret. A significant change was defined as >5% change and their differences in different treatment groups were tested for statistical significance using the Fisher’s exact test and non-parametric method (Wilcoxon-Mann-Whitney test).

### Ethics statement

All study procedures and animal cares were approved and conducted in accordance with the guidelines and under the supervision of Institutional Committee on Animal Care and Use, Institute of Preventive Medicine, National Defense Medical Center (approval # AV-102-27) following the Institutional Animal Care and Use Committee Guidebook published by the US Office of Laboratory Animal Welfare.

## Results

### Adaptation of NIBRG-268 in MDCK cells

After first passage in MDCK cells, the NIBRG-268 virus could only grow to about 10^5^ TCID_50_/ml (NIBRG268-M1). After three passages in MDCK cells, the NIBRG268-M3 virus could reach 10^7^ TCID_50_/ml and was then used to generate a master virus bank of 400 vials (HA titer 256, infectious virus titers: 3.16x10^7^ TCID_50_/ml and 2.20x10^7^ PFU/ml) following cGMP guidelines. Comparing nucleotide sequences of eight genes, the NIBRG268-M4 revealed a G/T polymorphism at 505 base of HA gene but NIBRG-M1 only revealed G at the same base. This single base change resulted in a Alanine/Serine polymorphism at 169 amino acid of HA. In addition, two synonymous mutations (C1369T HA gene and C1242T NA gene) are detected. Despite the polymorphism of the amino acid mutation in HA protein, the NIBRG268-M4 was confirmed to maintain similar antigenicity (<4-fold difference) with NIBRG268-egg by measuring HI antibody titers against the post-infection ferret antisera and standard antisera (US FDA lot # H7-Ab-1317). To confirm genetic stability of HA gene, the NIBRG268-M4 viruses were further cultured for 10 passages in MDCK cells. After 10 passages in MDCK cells, HA gene of the NIBRG268-M14 maintained 1 basic amino acid residue in the cleavage site, which is a genetic signature of low pathogenic avian influenza viruses.

### Production of vaccine antigens

**Fig 1:**
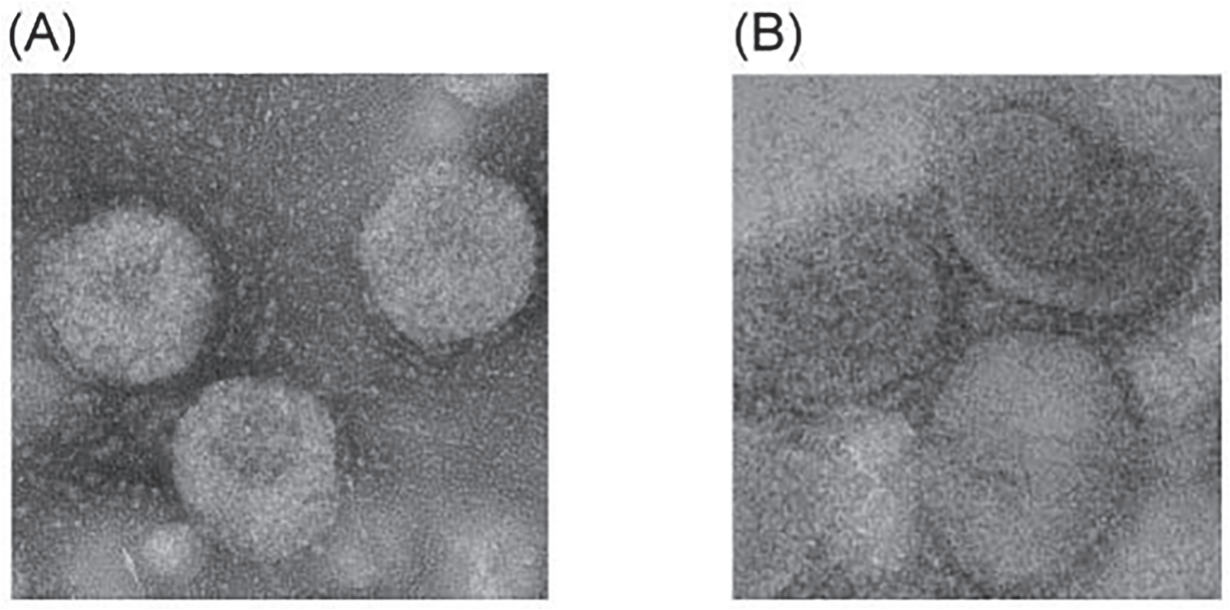
Electron micrograph (100,000×) of MDCK cell-derived (A) and egg-derived (B) influenza H7N9 virus particles after purification. Egg-derived influenza H7N9 virus particles were obtained from the US FDA as standard antigens of SRD assay.

Using 5 g/L of microcarriers, cells grew to confluence in microcarriers at day 5 or 6 and reached approximately 2×10^6^ cells/ml. After infecting NIBRG268-M4 viruses, virus titers reached approximately 10^7.5^ TCID_50_/ml at day 3. After purification, the bulk vaccine antigen contained whole virus particles which are similar to the egg-derived virus particles in morphology (Fig. 1).

### Immunogenicity in ferrets

Immunization regimens are shown in details in Table 1. Each group contained four ferrets (n = 4) for evaluating HI and neutralization titers at day 0 (prevaccination), day 14 (post dose 1) and day 21 (post dose 2). The GMT and their 95% confidence (95% CI) were calculated for comparison. Without adjuvant, the H7N9 vaccine antigen could not induce antibody responses in ferrets after the first dose of vaccination and only elicited low antibody response after the second vaccination (Table 1). After being adjuvanted with aluminum hydroxide, the H7N9 vaccine antigens could induce dose-dependent antibody response after one (HI GMT 48) and two (HI GMT 190) doses of vaccination using the HI assay, which are all significantly higher than that in the control and unadjuvanted groups. Similar results were observed using the neutralization assay (Table 1). Based on WHO surveillance data, the H7N9 viruses which have circulated in China since late 2013 are antigenically similar to A/Anhui/1/2013 strain. Therefore, only A/Anhui-like viruses have been used to generate H7N9 reassortant vaccine viruses and clinical trials using the A/Anhui-like vaccine viruses only evaluated antibody responses to the homologous vaccine viruses [25–27], which is similar to our study.

**Table 1:** Immunogenicity of inactivated influenza H7N9 whole virus vaccine in ferrets.

	Group 1	Group 2	Group 3
HA (μg/dose)	0	1.5	1.5
Al(OH)_3_ (μg/dose)	300	0	300
No. of animals	4	4	4
HI Seroconversion			
Prevaccination	0/4	0/4	0/4
Post dose 1	0/4	0/4	4/4
Post dose 2	0/4	4/4	4/4
HI GMT			
Prevaccination	<10	<10	<10
Post dose 1	<10	<10	48 (34–67)^a^
Post dose 2	<10	17 (9–33)	190 (98–396)
Nt Seroconversion			
Prevaccination	0/4	0/4	0/4
Post dose 1	0/4	0/4	4/4
Post dose 2	0/4	4/4	4/4
Nt GMT			
Prevaccination	<40	<40	<40
Post dose 1	<40	<40	48 (34–67)
Post dose 2	<40	80 (30–213)	640 (143–2189)

^a^Geometric mean titers (95% confidence interval)

### Protection in ferrets

**Fig 2:**
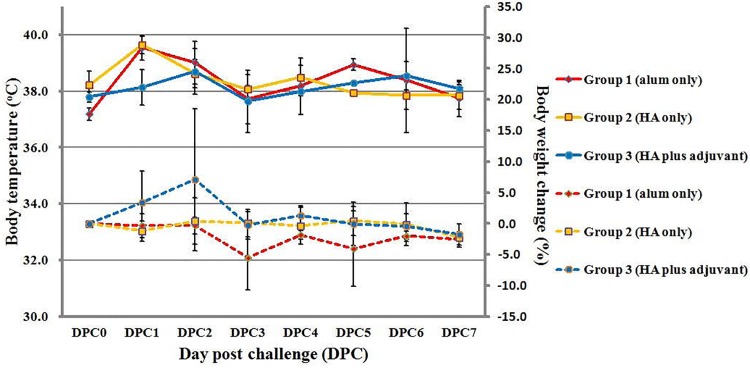
Changes in body temperatures and weights in ferrets within 7 days post challenge (DPC) with wild-type influenza H7N9 virus. The solid and dotted lines represent changes of body temperatures and weights, respectively. Data are expressed as a percentage of the baseline and the vertical bars represent group standard deviations (mean ± SD). 0–3 DPC: 4 ferrets for each group; 4–7 DPC: 2 ferrets for each group.

No significant side effect and reactogenicity were observed in the three treatment groups during the 7 days postvaccination period after the first and second doses. Within 7 days post challenge, no clinical symptoms could be observed in the three treatment groups. However, two ferrets in the control group developed significant body weight loss (body weight loss: -5.9%, -12.7%, -1.9%, -1.6%) at 3 DPC. In contrast, no ferret in the unadjuvanted (body weight loss: 3.2%, -2.0%, -0.9%, 0.2%) and adjuvanted (body weight loss: -2.9%, 1.3%, -1.2%, 1.9%) groups developed significant body weight loss (P = 0.06, one side Fisher’s exact test; P = 0.04, one side Wilcoxon-Mann-Whitney test) at 3DPC (Fig. 2). For body temperature change, ferrets in control and unadjuvanted groups revealed mild increase in body temperature change at 2 DPC than that in adjuvanted group. However, no ferret in the three treatment groups had significant temperature increase (defined as temperature increase >5%) (Fig. 2).

**Fig 3:**
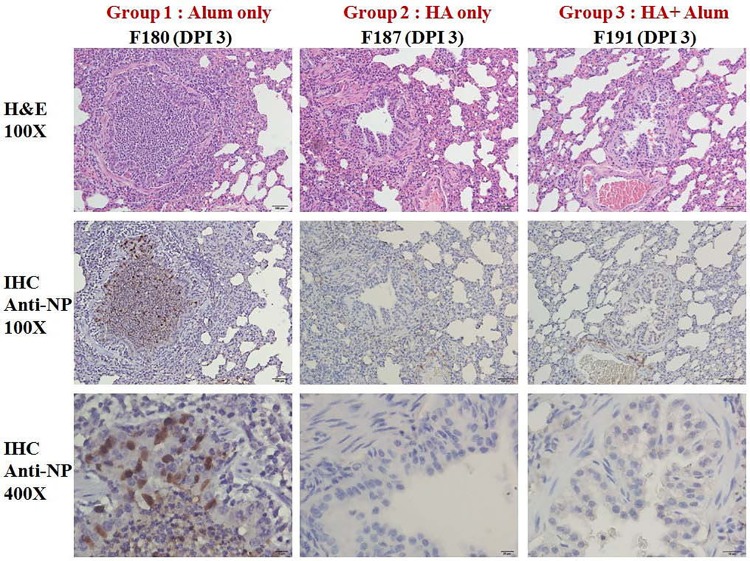
Histopathological examination and immunohistochemical (IHC) staining in lung tissues of ferrets at 3 days post challenge (DPC) with wild-type influenza H7N9 viruses. Top panel is H&E staining with 100-fold magnification (100×), middle panel is IHC staining with 100×, and bottom panel is IHC staining with 400×. Tissue with brown color indicates viral antigen detected by a mouse anti-NP monoclonal antibody in IHC staining.

Regarding virus replication, virus RNA copies in the control group were detected in multiple organs at day 3 post challenge and were more likely to be detected in nasal turbinates at day 7 post challenge. Similar patterns were observed for measuring live virus titers in the control group. In contrast, low virus RNA copies were detected in the unadjuvanted and adjuvanted vaccine groups, but live viruses were only detected in the unadjuvanted group (Table 2). In addition, histopathology and IHC staining in lung tissues of ferrets at 3 DPC were conducted by microscopic examination. Histopathological examination showed that acute inflammatory cells (neutrophils) and cellular debris infiltrated in the bronchioles and alveoli of lungs in the control group. In IHC staining, viral antigen-positive (brown color) cells were also detected in the epithelial cells of bronchioles in the control group. However, viral antigen-positive cells could not be detected in the unadjuvanted and adjuvanted vaccine groups (Fig. 3). Overall, the adjuvanted H7N9 vaccine is highly immunogenic and could reduce virus replication against wild-type influenza H7N9 virus challenge in ferrets.

**Table 2:** Virus titers in vaccinated ferrets at 3 and 7 days post challenge (DPC) with wild-type influenza H7N9 virus.

	Group 1		Group 2		Group 3	
	Virus RNA copies		Virus RNA copies		Virus RNA copies	
	(live virus titers)^a^		(live virus titers)^a^		(live virus titers)^a^	
Ferret ID	#180	#188	#184	#187	#181	#191
3 DPC						
Nasal turbinate	6.3 (5.6)	6.5 (2.2)	5.9 (2.6)	6.2 (2.3)	3.7 (<1)	4.3 (<1)
URT	<2 (ND)	4.2 (1.9)	<2 (ND)	4.3 (<1)	<2 (ND)	<2 (ND)
LRT	2.4 (ND)	4.5 (3.3)	<2 (ND)	5.1 (<1)	<2 (ND)	<2 (ND)
HLN	<2 (ND)	2.5 (ND)	<2 (ND)	2.0 (ND)	<2 (ND)	<2 (ND)
Lung	2.3 (ND)	<2 (ND)	<2 (ND)	<2 (ND)	<2 (ND)	<2 (ND)
Ferret ID	#194	#197	#190	#199	#198	#201
7 DPC						
Nasal turbinate	5.9 (2.5)	4.2 (<1)	3.3 (<1)	2.3 (<1)	2.4 (<1)	<2 (<1)
URT	<2 (ND)	<2 (ND)	<2 (ND)	<2 (ND)	<2 (ND)	<2 (ND)
LRT	2.8 (ND)	<2 (ND)	<2 (ND)	<2 (ND)	<2 (ND)	<2 (ND)
HLN	2.4 (ND)	<2 (ND)	<2 (ND)	<2 (ND)	<2 (ND)	<2 (ND)
Lung	<2 (ND)	<2 (ND)	<2 (ND)	<2 (ND)	<2 (ND)	<2 (ND)

^a^Data are presented as log_10_/mg and quantification limits for virus RNA copies and live virus titers are 100 copies/mg and 10 TCID_50_/mg, respectively.

URT: upper respiratory tract (top 1/3 of trachea); LRT: lower respiratory tract (bottom 1/3 of trachea); HLN: hilar lymph node; ND: not determined.

## Discussion

Based on online registrations (www.clinicaltrials.gov/) and reports from the European Center for Disease Prevention and Control (www.ecdc.europa.eu), as of November 2014 as total of thirteen clinical trials of influenza H7N9 vaccines have been conducted (Table 3). Among them, three clinical trials used egg-derived live-attenuated vaccines, four used egg-derived split vaccines, one used recombinant H7 DNA vaccine, two used inactivated A/Shanghai/2/2013 (H7N9) vaccines, two used recombinant virus-like particle (VLP) vaccines, and one used MDCK cell-based inactivated subunit vaccine, which were all different from our vaccine candidate (MDCK cell-derived inactivated whole virus vaccine). Two clinical studies, including one VLP vaccine and one MDCK-derived subunit vaccine, have been completed and published. The influenza H7N9 VLP vaccine study was conducted in healthy Australian adults and evaluated two doses of VLP vaccine antigens with and without adjuvant. The influenza H7N9 VLP vaccine was not immunogenic at low and high HA dosage level (15 vs. 45μg of HA) without adjuvant (HAI antibody seroconversion rates 5.7% vs. 15.6%) but was highly immunogenic when adjuvanted with saponin-based ISCOMATRIX (60 units) at low and intermediate HA dosage level (5 vs. 15μg of HA) (HAI antibody seroconversion rate 80.6% vs. 64.7%) [26]. Interestingly, the VLP vaccine candidate without adjuvant was highly immunogenic in mice, which is inconsistent with the human study [28]. The MDCK cell-based inactivated subunit vaccine study conducted in healthy US adults evaluated two doses of vaccine with and without adjuvant (MF59). The MDCK cell-based subunit vaccine at 15μg of HA dosage level was not immunogenic without adjuvant but was highly immunogenic when adjuvanted with MF59 (HAI antibody seroconversion rates 8% vs. 85%) [25]. Animal studies of the MDCK cell-derived influenza H7N9 vaccine have not been published and it would be desirable to compare animal and human studies to identify suitable animal models for evaluating pandemic influenza vaccines with different formulations.

**Table 3:** Clinical trials of influenza H7N9 vaccines until July 2014.

Clinical Trial No.	Brief Title	Sponsor	Vaccine Type
1. NCT02151344	Evaluating the safety and immune response to a live H7N9 influenza virus vaccine followed by an inactivated H7N9 influenza virus vaccine, given at varying intervals	NIAID^a^	Egg-derived live attenuated A/Anhui/13 ca virus (H7N9) virus vaccine
2. NCT01995695			
3. NCT02274545			
4. NCT01938742	H7N9 mix and match with AS03 and MF59 in healthy adults	NIAID	Egg-derived A/Shanghai/2/2013 (H7N9) split vaccine
5. NCT01942265			
6. NCT02251288			
7. NCT02213354			
8. NCT02206464	Recombinant H7 DNA plasmid Vaccine, VRC-FLUDNA071-00-VP, administered alone or with monovalent influenza subunit virion H7N9vaccine (MIV)	NIAID	Recombinant H7 DNA plasmid vaccine
9. NCT01999842	Immunogenicity and safety study of GlaxoSmithKline (GSK) Biologicals’ influenza vaccines GSK3206641A, GSK3206640A, GSK3277510A and GSK3277509A in adults 18 to 60 years of age	GSK	Inactivated A/Shanghai/2/2013 (H7N9) vaccine
10. NCT02177734			
11. NCT01897701	A/H7N9 Virus-Like Particle (VLP) antigen dose-ranging study with Adjuvant 1 or Matrix-M1 adjuvant	Novavax	Monovalent A/Anhui/1/13 (H7N9) VLP
12. NCT02078674			
13. NCT01928472	Dose-finding study of four dosage levels of an H7N9 influenza vaccine in adults between ages of 18 Years and 65 Years	Novartis Vaccines	Cell-derived (MDCK) inactivated monovalent subunit H7N9 virus vaccine

^a^NIAID: National Institute of Allergy and Infectious Diseases

It is well documented that ferrets are suitable animals for evaluating pathology and transmission of influenza viruses [6, 29]. However, no standard protocol regarding challenge intervals has been proposed for evaluating immunogenicity and protection of human influenza vaccines in ferrets, which varied significantly in different studies using different formulations [19, 30–32]. The interval between the last vaccination of inactivated influenza vaccines and virus challenge varied from 1 to 4 weeks in different studies [19, 31, 32]. In our study, we selected a 2-week interval for virus challenge and may not exclude the possibility of non-specific innate immune responses contributing to the protection observed in ferrets, which will need further studies to clarify. Besides, it is important to select relevant dosages and formulations for predicting human antibody responses but also no international guidance about the dosage and formulation design is available. In our study, we selected 1/10 of seasonal influenza vaccine dosage (15μg of HA protein) for evaluating MDCK cell-based whole virus antigens adjuvanted with aluminum hydroxide in ferrets. Based on antibody responses in our study, immunogenicity of the whole virus vaccine antigens was suboptimal but could be significantly improved when adjuvanted with aluminum hydroxide. Further studies of the adjuvanted vaccine candidates with different HA dosages in humans are warranted.

Current egg-based technology could produce about one billion doses of seasonal trivalent influenza vaccines annually but only 500–600 million doses were supplied due to low demand. The egg-based technology requires 6 months to prepare a large amount of embryonated eggs and cannot promptly meet the surging global demand during an influenza pandemic [9]. This was illustrated in the 2009 H1N1 pandemic where only 22% of expected doses were supplied within the first 6 months after the pandemic was declared [8]. Moreover, egg-based manufacturing capacity could be vulnerable during pandemics caused by highly pathogenic avian influenza viruses (HPAIV) which could kill hens rapidly. Therefore, vaccine production using cell-based technology is becoming an attractive alternative. Our study supports the feasibility of manufacturing influenza H7N9 vaccines in a microcarrier-based MDCK cell culture system.

Currently, the WHO reference laboratories only generate egg-derived high growth reassortant influenza H7N9 vaccine viruses. Therefore, we need to adapt the egg-derived high growth vaccine viruses in MDCK cells to increase growth efficiency, which is time consuming. It would be desirable to establish MDCK cell-derived high growth master donor virus and generate high growth reassortant vaccine viruses for pandemic preparedness through the WHO network. In addition, adjuvant development is also critical to meet surging demand during an influenza pandemic. Due to commercial considerations, we included a traditional alum adjuvant in our study and the alum adjuvant boosted immunogenicity of the influenza H7N9 whole virus antigens in ferrets. It would be desirable to compare different antigen types and adjuvants in ferrets to select the most promising influenza H7N9 vaccine candidates for clinical trials.

Three nucleotide mutations (2 in HA gene and 1 in NA gene) including one single amino acid change related to the Alanine169Serine polymorphism of HA gene were detected in the NIBRG268-M4 virus which has a better growth efficiency in MDCK cells than the NIBRG268-egg in the present study. The molecular determinants of the MDCK cell-adapted high growth NIBRG268 virus are unknown at the moment, which could be elucidated using reverse genetics in the future.

In conclusion, avian-origin influenza H7N9 viruses are circulating widely in China and pose increasing threats to global health. Development of influenza H7N9 vaccines for pandemic preparedness is recommended, especially for countries neighboring China. In this study, we have demonstrated immunogenicity and protection in ferrets of the MDCK cell-derived influenza H7N9 whole virus vaccine adjuvanted with aluminum hydroxide. Clinical trials are warranted to evaluate this vaccine candidate.
